# The Implications of the Brønsted Acidic Properties of Crabtree-Type Catalysts in the Asymmetric Hydrogenation of Olefins

**DOI:** 10.1021/jacs.2c07023

**Published:** 2022-08-31

**Authors:** Bram B. C. Peters, Pher G. Andersson

**Affiliations:** † Department of Organic Chemistry, Stockholm University, Svante Arrhenius väg 16C, SE-10691 Stockholm, Sweden; ‡ School of Chemistry and Physics, University of Kwazulu-Natal, Private Bag X54001, Durban, 4000, South Africa

## Abstract

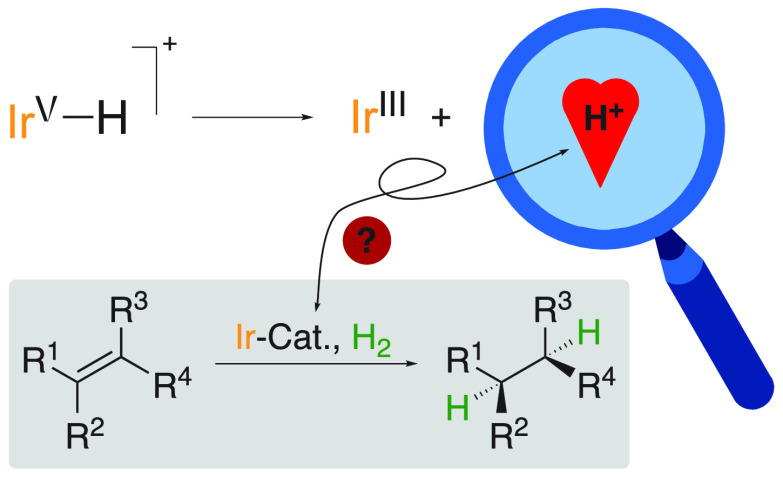

Chiral iridium complexes derived from Crabtree’s catalyst are highly useful in modern hydrogenations of olefins attributed to high reactivity, stereoselectivity, and stability. Despite that these precatalysts are pH neutral, the reaction mixtures turn acidic under hydrogenation conditions. This Perspective is devoted to the implications of the intrinsic Brønsted acidity of catalytic intermediates in asymmetric hydrogenation of olefins. Despite that the acidity has often been used only as a rationale for side-product formation, more recent methodologies have started to use this property advantageously. We hope that this Perspective serves as a stimulant for the development of such compelling and new asymmetric hydrogenations. The inherent scientific opportunities in utilizing or annihilating the generated Brønsted acid are enormous, and potential new innovations are outlined toward the end.

## Introduction

**Figure 1:**
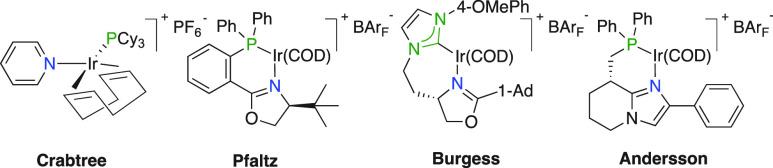
Crabtree’s catalyst and chiral analogs.

Catalytic asymmetric hydrogenation of olefins constitutes an indispensable tool for the installation of stereogenic centers in prochiral material. Highlighted by awarding half of the Nobel Prize in Chemistry in 2001^1^ to Knowles and Noyori (Rh and Ru catalyzed hydrogenation, other half awarded to Sharpless), transition-metal catalyzed asymmetric hydrogenation attracts both academic and industrial research due to generally high atom economy and stereoselectivity.^2^ Apart from the advances attained using Rh and Ru catalysis, chiral analogs of Crabtree’s catalyst ([(COD)IrPCy_3_(py)]PF_6_) (Figure 1), which have been developed over the past three decades, are ubiquitous in transition-metal catalyzed hydrogenations. Together, these three metals currently dominate the field of catalytic asymmetric hydrogenation of olefins.^3^ The first chiral analogs introduced by Pfaltz consists of a bidentate PHOX ligand derived from readily available amino acids.^4^ In a successive study, the PF_6_ counteranion was replaced by the more weakly coordinating BAr_F_ anion that suppressed irreversible trimerization of catalytic intermediates, deactivating the catalyst.^5^ These findings laid the foundation for further development of asymmetric hydrogenations of olefins using iridium-based catalysts. Another noteworthy and efficient class of ligands was developed by Burgess where the phosphorus coordination side was replaced by a N-heterocyclic carbene (NHC).^6^ Our group has also contributed to the design and evaluation of structurally diverse N,P ligated iridium complexes.^7^ Presently, still a continuously vast study on the preparation and evaluation of novel chiral Crabtree-type complexes is being carried out.

**Figure 2:**
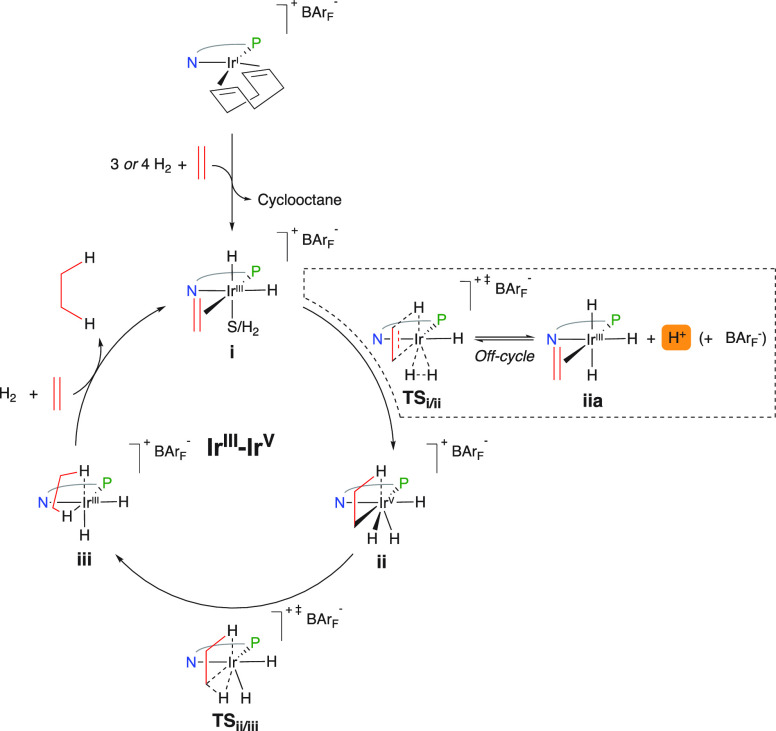
Proposed catalytic cycle (Ir^III^–Ir^V^) of the iridium-catalyzed hydrogenation of unfunctionalized olefins. S = solvent.

Concomitant with the development of new iridium complexes, mechanistic studies have been carried out to elucidate hydrogenation pathways.^8^ The most widely accepted mechanism for the hydrogenation of unfunctionalized olefins by Crabtree-type complexes involves an Ir^III^–Ir^V^ cycle (Figure 2). The iridium-based precatalyst reacts rapidly with hydrogen gas to produce cyclooctane, and the catalytically active dihydride species **i** forms. An unfunctionalized olefin then preferentially coordinates in *trans* position to phosphorus as suggested on the basis of computational studies. Subsequent rate- and selectivity-determining migratory insertion into the Ir–H bond occurs concurrent with the oxidative addition of a ligated molecular hydrogen forming transient Ir^V^-intermediate **ii**. The alkane is then liberated by reductive elimination (species **iii**) and successively displaced by a new olefin and H_2_ to close the catalytic cycle. Most important, experimental support for this hypothetical mechanism was reported by Pfaltz where NMR experiments elucidated an Ir^III^ dihydride alkene complex (akin to intermediate **i**), proposing the resting state and the necessity of molecular hydrogen to proceed.^9^

**Figure 3:**
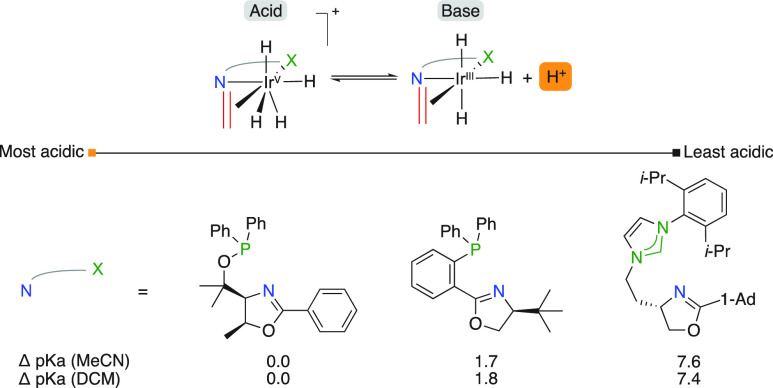
Calculated relative Brønsted acidity of NHC,N and N,P ligated catalytic intermediates.

All the individual starting components for hydrogenation (the precatalyst, the olefin, and molecular hydrogen) are pH neutral; however, this is not true for the catalytic intermediates. The intrinsic Brønsted acidic property arises from an off-cycle event where a transient Ir^V^-intermediate releases a proton and forms a neutral Ir^III^-species **iia**. Consequently, the electronic properties of the chiral ligand have a large impact on the acidic feature of these catalytic intermediates and several studies have elucidated structural aspects in projection of acidity. The first groundbreaking study by Burgess in 2010 initially described the acidic phenomena of catalytic intermediates.^10^ A DFT study was undertaken to compute the relative p*K*_a_ values of N,P- and N,NHC-ligated complexes using the Born–Haber cycle starting from cationic tetrahydride-Ir^V^-ethylene intermediates (Figure 3). The outcome was significant, revealing a large difference of 7 p*K*_a_ units with the N,P complexes being more acidic. A self-explanatory experiment using an ordinary pH indicator (methyl red) also demonstrated the pronounced differences between different catalysts. The easier dissociation of a proton in phosphorus ligated intermediates over the NHC counterparts was rationalized by two amplifying effects: (1) NHCs exhibit strong σ-donating ability, being superior in stabilizing the Ir^V^ intermediate and (2) are inferior π-acceptors hence stabilizing the Ir^III^ species to a lesser extent.

**Figure 4:**
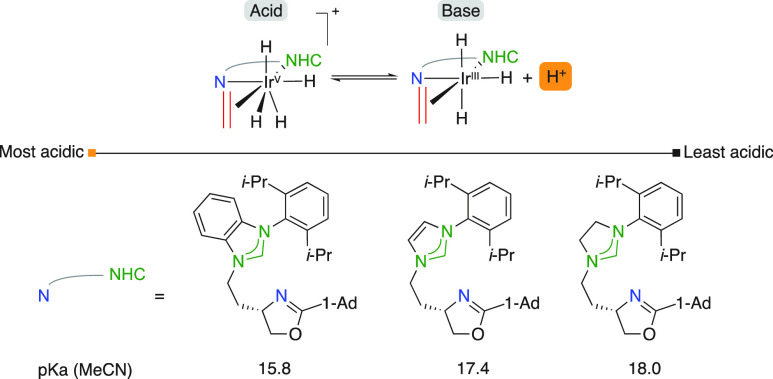
Calculated Brønsted acidity of NHC,N ligated catalytic intermediates.

In a successive study, Burgess compared the acidity of three different NHC backbones and found descending acidity among the benzimidazolylidene, imidazolylidene and imidazolinylidene backbones (Figure 4).^11^ The σ-donor and π-acceptor potentials were used to rationalize the calculated trend.

**Figure 5:**
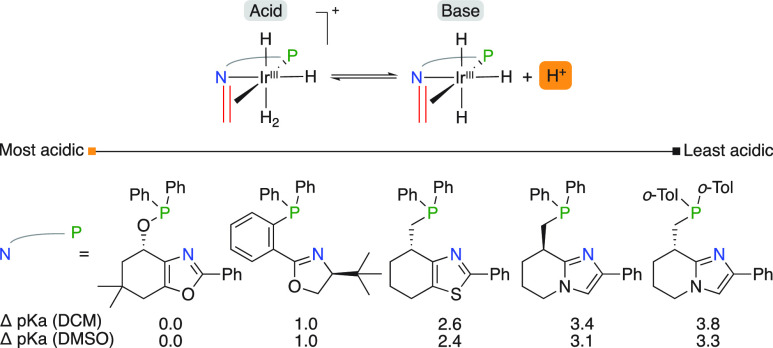
Calculated relative Brønsted acidity of N,P ligated catalytic intermediates.

The Andersson group also performed similar acidity calculations on their iridium-based N,P complexes (Figure 5).^12^ It was found that the acidity of the catalytic intermediate was influenced by the heterocycle in the backbone (up to 3.8 p*K*_a_ units) and followed the same trend as postulated for the acidity of unsubstituted and protonated heterocycles.

Despite that the Brønsted acidic feature of catalytic intermediates in the iridium-catalyzed hydrogenation of olefins is widely accepted at present, and is used to rationalize side-product formation in the hydrogenation of olefins bearing acid labile groups, it is to a lesser extent embraced and taken into advantage for the development of novel asymmetric hydrogenations. Although there are several published reviews on the Brønsted acid strength of metal hydrides and dihydrogen complexes, the implications in asymmetric methodology development remain less studied.^13^ This perspective aims to shed light on the advantageous feature of using, or neutralizing, the generated Brønsted acid. The application of these compelling new methodologies in the synthesis of natural products is also highlighted. As a last note, iridium-based catalysts (typically involving an Ir^III^–Ir^V^ cycle) are of particular interest for this purpose since higher oxidation states are accessed in comparison to rhodium and ruthenium (typically involving a M^I^–M^III^ cycle) which result in more electron-deficient metal centers. For example, Burgess calculated Crabtree’s catalyst to be 25 p*K*_a_ units more acidic (in MeCN) than Wilkinson’s catalyst (RhCl(PPh_3_)_3_).^10^

## BrØnsted Acidity as a Rationale for Reactivity and/or Side-Product Formation

Chiral analogs of Crabtree’s iridium complex are efficient precatalysts for a host of alkene hydrogenations, often yielding the desired reduced alkane in quantitative yield. However, in some cases side-product formation was observed during the hydrogenation of vinyl functionalized olefins. Particularly substrates bearing acid labile groups sometimes formed complex mixtures of products and, depending on the ligand backbones, could even lead to complete decomposition. The following section highlights selected observations, focusing on structural aspects in the used complexes in projection of reactivity and product selectivity.

**Figure 6:**
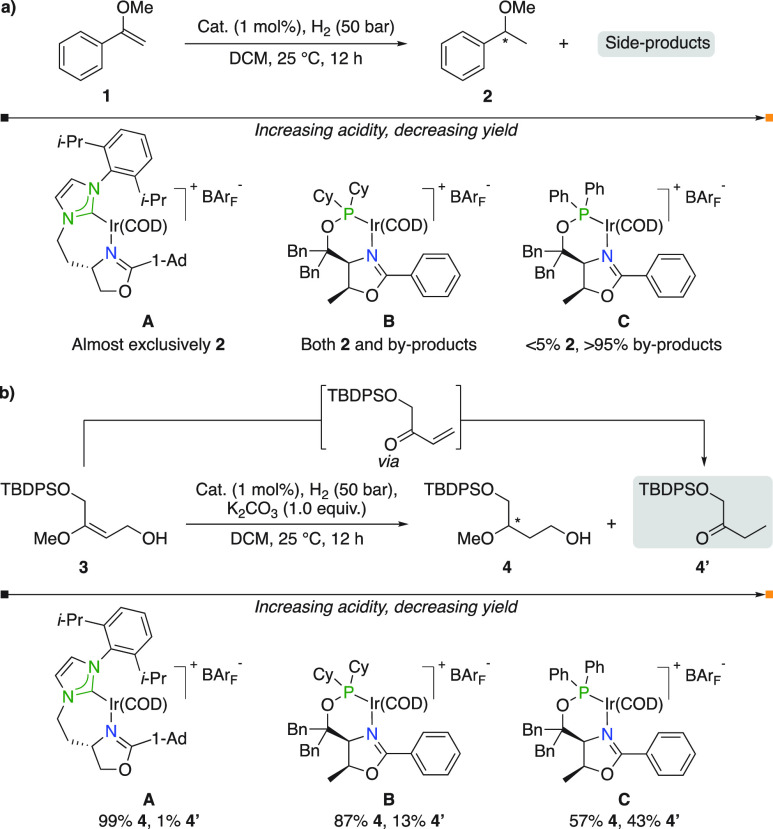
(a) Hydrogenation of aromatic enol ether **1**. (b) Hydrogenation of aliphatic enol ether **3**.

To support the calculated acidity difference between N,P- and N,NHC-ligated catalytic intermediates, Burgess evaluated the corresponding catalysts in the hydrogenation of acid-sensitive enol ether **1** (Figure 6a).^10^ The reaction proceeded smoothly, producing almost exclusively the desired product **2** when N,NHC complex **A** was used. On the other hand, a large quantity of side-products was formed when the hydrogenation was catalyzed by the more acidic N,P complexes and <5% of the desired product was obtained in the case of catalyst **C**. The observed trend agreed with the computed acidities; however, quantification and characterization of the side-products was found to be difficult. Therefore, the same set of catalysts were evaluated against the hydrogenation of enol ether **3** that, upon acid mediated elimination of water and subsequent hydrogenation of the formed enone, can lead to ketone **4′** (Figure 6b). Ascendingly more **4′** was formed when the more acidic catalysts were used, strengthening the computational findings.

**Figure 7:**
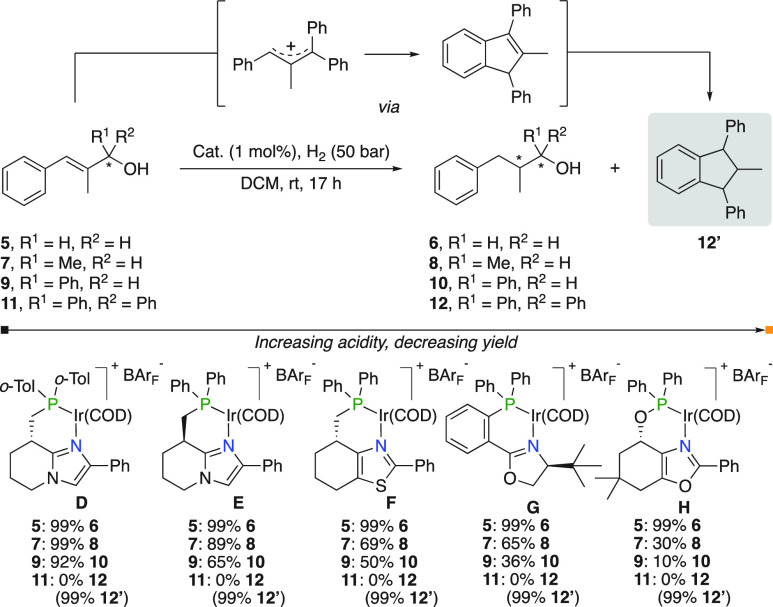
Hydrogenation of allylic alcohols.

The Andersson group reported a matrix consisting of the hydrogenation of different allylic alcohols by five structurally diverse N,P complexes (Figure 7).^12^ Catalysts **D** to **H** were postulated to produce progressively more Brønsted acid under hydrogenation conditions, and a clear trend in reactivity as an effect of their intrinsic Brønsted acidity emerged. Primary allylic alcohol **5** was efficiently reduced to **6** in 99% yield by all catalysts. However, when the carbinol carbon was substituted with a methyl or phenyl group (**7** and **9**, respectively), the formation of the desired product was reduced upon the use of more acidic catalysts. This was explained as a consequence of the formation of a more stable carbocation upon protonation of the allylic alcohol. For tertiary allylic alcohol **11**, no product was obtained in all cases and instead an intramolecular Friedel–Crafts type alkylation and consecutive hydrogenation of the formed indene was observed. Indene formation starting from **11** was previously described to occur in the presence of Brønsted or Lewis acids.^14^

**Figure 8:**
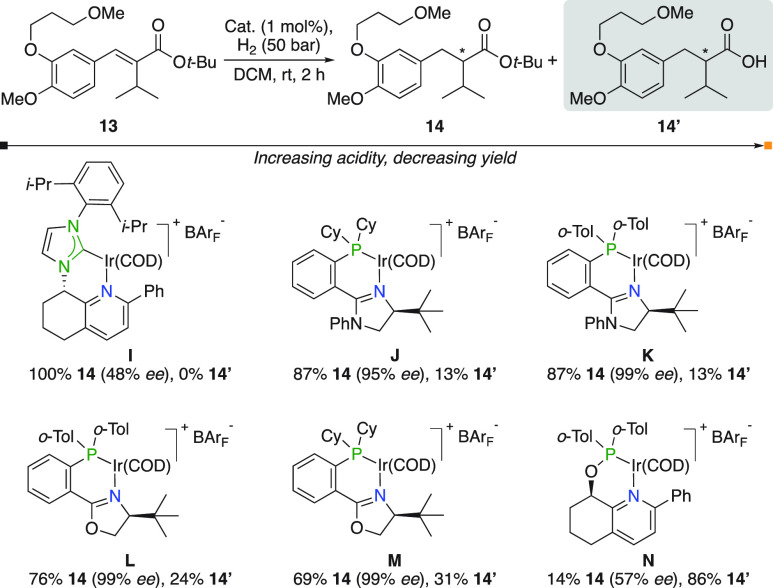
Hydrogenation of unsaturated *tert*-butyl ester.

Distributed over two publications, Pfaltz observed the formation of carboxylic acid **14′** in the hydrogenation of α,β-unsaturated *tert*-butyl ester **13**, an intermediate in the synthesis of renin-inhibitor Aliskiren (Figure 8).^15,16^ The more basic NHC,pyridine complex **I** was found to be superior and produced solely **14**, whereas all evaluated N,P catalysts yielded variable amounts of **14′**.

In addition to what has been mentioned above, there exists numerous reports on reactivity differences between iridium catalysts as a function of their acidities, or the acid promoted formation of side-products.^17−27^ We recommend consulting individual studies for specific details.

## Using the Acidity for the Development of New Methodology

This Perspective has so far dealt with the generated Brønsted acid under hydrogenative conditions from a viewpoint of producing side-products. The enormous potential in using the acidic environment for the development of new reactions is at present date only scratching the surface. The following section describes the methodologies that used the generated acid, resulting in the development of compelling asymmetric hydrogenations.

### In Situ Olefination

Most iridium-catalyzed hydrogenations require starting from isomerically pure olefins since the double bond isomerism normally governs the stereochemical outcome.^28^ Traditional olefination methods often produce isomeric mixtures that thus require tedious and waste-producing separation prior to hydrogenation. Racemic alcohols, on the other hand, are easily prepared by a Grignard reaction and are often more easily isolated from the reaction mixture compared to other olefinations (for example the Wittig reaction produces stoichiometric amounts of waste containing phosphorus). Owing to the lability of hydroxyl groups under hydrogenation conditions, the elimination of water provides the opportunity for in situ olefination.

**Figure 9:**
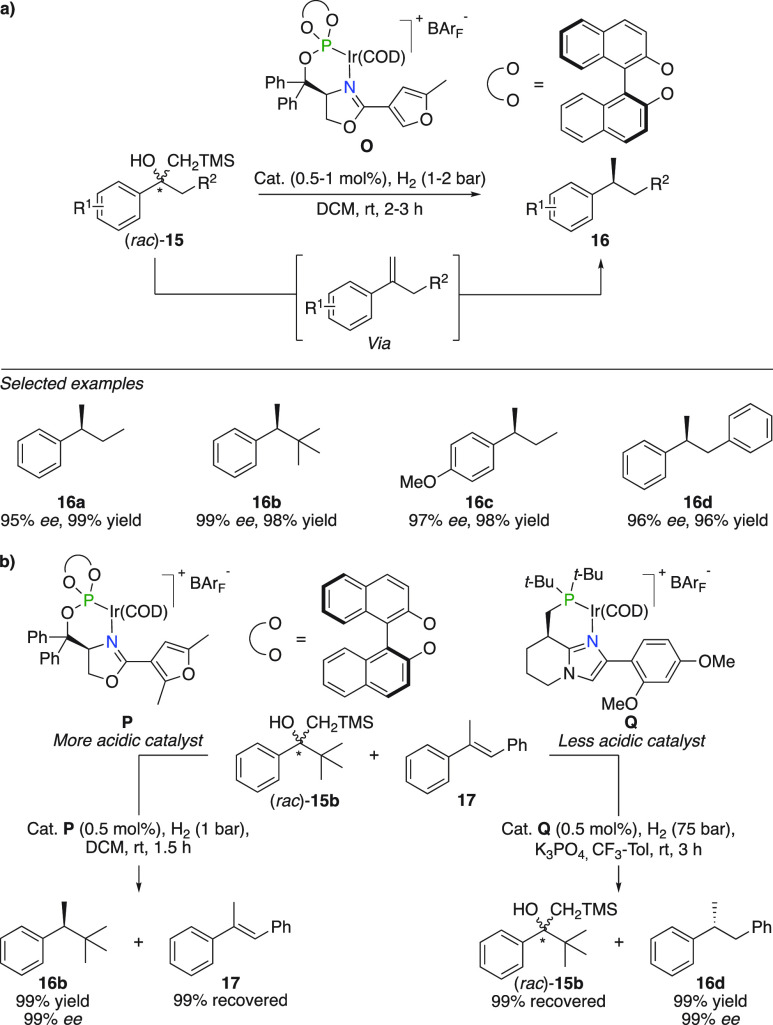
Tandem Peterson elimination and hydrogenation. (a) Selected examples. (b) Competition experiments.

The first published report using this strategy concerned the acid-catalyzed Peterson elimination of β-hydroxysilanes that formed a disubstituted alkene prior to hydrogenation (Figure 9a).^29^ Substrates bearing variations on the aromatic ring as well as different side-chains connected to the benzylic carbon efficiently underwent Peterson elimination followed by hydrogenation using this method (**15a**–**d**). To demonstrate the involvement of a Brønsted acid in the elimination reaction, competition experiments using β-hydroxysilane (*rac*)-**15b** and α-methyl stilbene **17** were carried out (Figure 9b). In a first experiment employing 0.5 mol % of catalyst **P** under 1 bar hydrogen atmosphere, β-hydroxysilane (*rac*)-**15b** was completely consumed whereas 99% of the unreacted α-methyl stilbene **17** was recovered. A second hydrogenation with 0.5 mol % of catalyst **Q** and 75 bar hydrogen in the presence of K_3_PO_4_ resulted in an antithetical outcome; **17** was completely reduced to **16d** whereas 99% of the β-hydroxysilane (*rac*)-**15b** was recovered.

**Figure 10:**
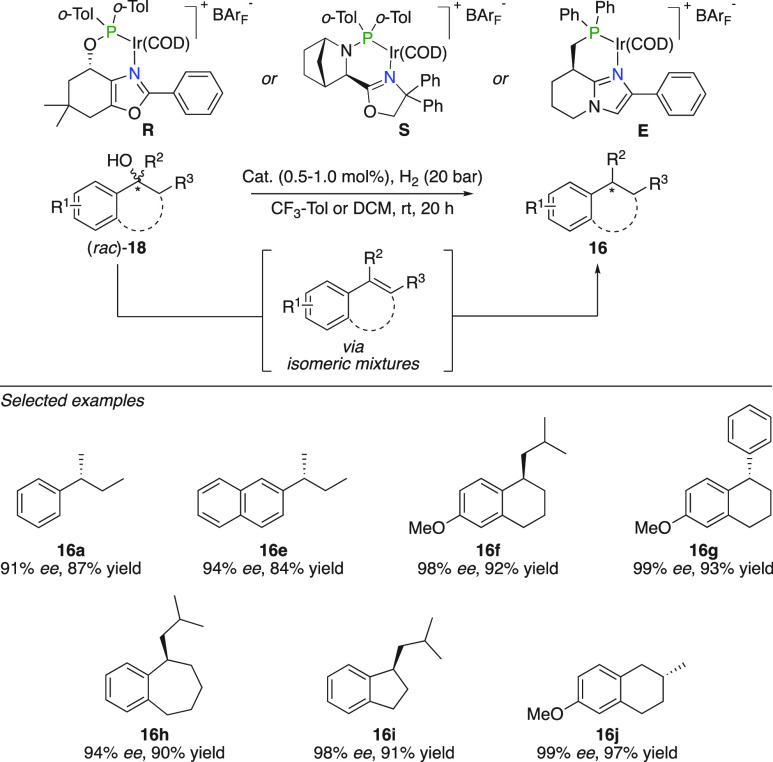
Formal deoxygenation of racemic alcohols.

The above-mentioned β-hydroxysilanes can react in a predictable manner to form the terminal disubstituted alkene. Tertiary alcohols on the other hand potentially undergo elimination to produce numerous isomeric olefins. Despite this, the Andersson group reported the formal enantioselective deoxygenation of benzylic alcohols yielding the corresponding alkane in considerably high enantioselectivities (Figure 10).^30^ A number of acyclic and cyclic benzylic alcohols were efficiently deoxygenated to provide the alkane in enantiomeric purity up to 99% *ee* (**16a**,**e**–**j**).

### Spiroketalization

**Figure 11:**
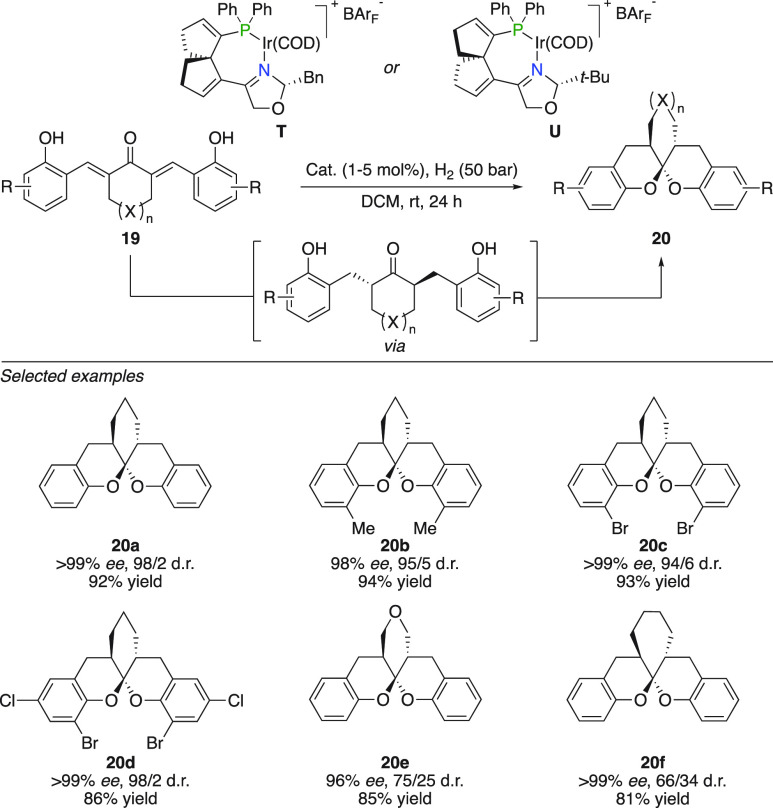
Hydrogenation and spiroketalization of α,α′-bis(2-hydroxyarylidene) ketones.

Wang and Ding reported the hydrogenation of α,α′-bis(2-hydroxyarylidene) ketones catalyzed by a spinPHOX ligated iridium complex (Figure 11).^31^ Instead of yielding 2,6-disubstituted cyclohexanones, the optically active intermediate cyclized under the reaction conditions and led to the formation of chiral aromatic spiroketals. Various substitutions on the phenol were tolerated as well as tetrahydropyran-4-one and cycloheptanone derived analogs (**20a**–**f**).

The involvement of Brønsted acid or Lewis acidic iridium species in the spiroketalization was not completely discriminated, since both had previously been reported to catalyze spiroketonalizations of these types of motifs.^32^ However, when the intermediate product was treated with the precatalyst under inert atmosphere, no reaction occurred. As soon as the atmosphere was exchanged to hydrogen gas, spiroketalization occurred. Other Ir^I^ based catalysts also did not catalyze the cyclization while IrCl_3_·3H_2_O did. This indicated that the spiroketalization was either catalyzed by a Brønsted acid, a high-valent iridium species, or both.

### Dynamic Kinetic Resolution

**Figure 12:**
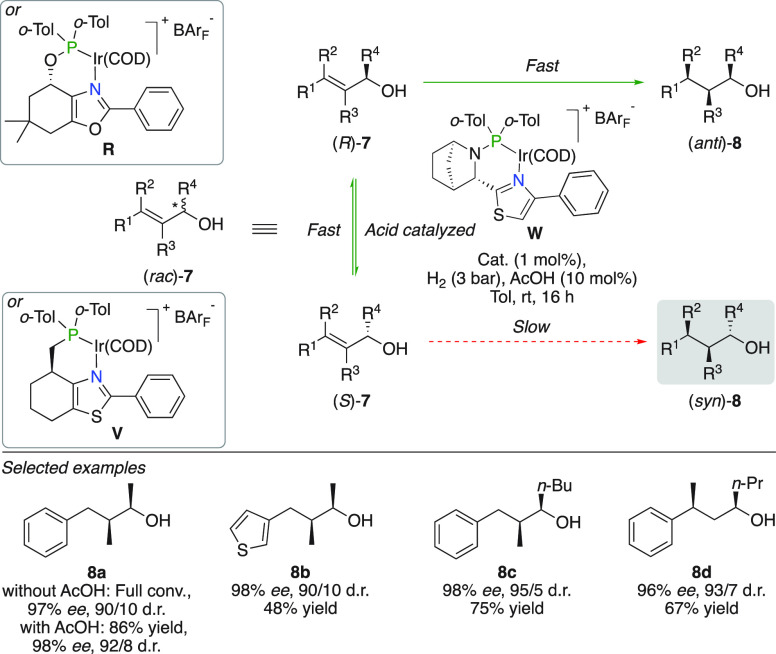
Dynamic kinetic resolution of allylic alcohols.

The aforementioned sections have already described the lability of allylic alcohols under hydrogenation conditions. The Andersson group realized that the generated Brønsted acid can be exploited for fast racemization of starting material and, accompanied by a large rate difference in hydrogenation of both enantiomers, the dynamic kinetic resolution of secondary allylic alcohols was established (Figure 12).^33^ Although good results were already obtained without additives (97% *ee*, 90/10 d.r. of **8a**), addition of 10 mol % AcOH was found to be beneficial to further accelerate the isomerization of the starting allylic alcohol to slightly improve the stereoselective outcome (98% *ee* and 92/8 d.r.). The protocol was tolerant to numerous substrates including heterocyclic substituted allylic alcohols, variations of the carbinol substituent, and β-prochiral equivalents (**8a**–**d**).

## Neutralizing the Acidity for the Development of New Methodology

The preceding part described methodologies that utilized the generated acid for the development of new hydrogenations. On the contrary, hydrogenation sometimes benefits by neutralizing the formed Brønsted acid. Two protocols that rely heavily on exclusion of acid are discussed in the following.

### Kinetic Resolution

**Figure 13:**
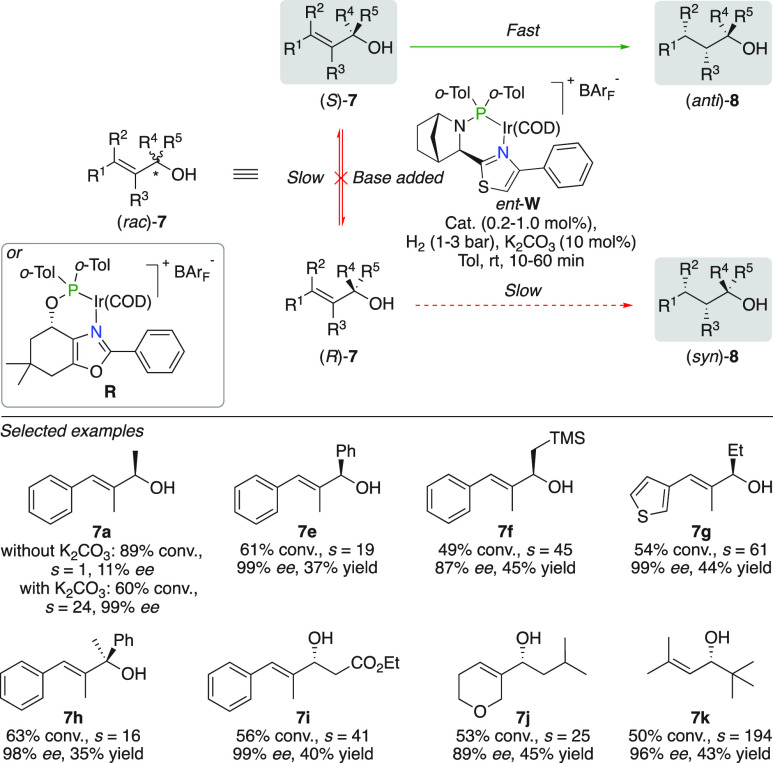
Kinetic resolution of allylic alcohols.

The racemization of chiral allylic alcohols under hydrogenation conditions was used advantageously for dynamic kinetic resolution. In contrast to this strategy, Andersson reported the highly efficient kinetic resolution of a wide variety of allylic alcohols (Figure 13).^34^ Critical to success was the addition of K_2_CO_3_ to neutralize the generated Brønsted acid during the hydrogenation, eradicating epimerization of the starting material. The large difference in reactivity between the employed chiral iridium complex and both enantiomers of the allylic alcohols accounts for minimal consumption of the desired enantiomer with *s*-values up to 211. It was demonstrated that a kinetic resolution was not established in the absence of K_2_CO_3_. Using the optimized conditions, a large number of secondary and tertiary allylic alcohols including benzylic alcohols, sensitive β-hydroxysilanes, heterocyclic substitutions, functionalized side-chains, and purely alkyl substituted substrates were efficiently resolved (**7a,e**–**k**).

### Desymmetrization

**Figure 14:**
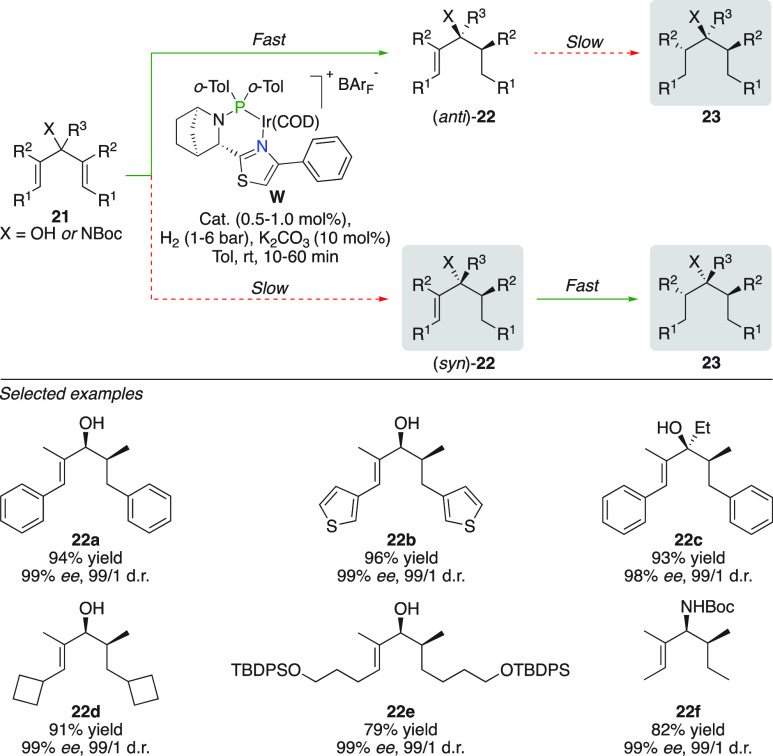
Desymmetrization of divinyl carbinols and carbinamides.^36^

The methodology was extended to the development of an efficient desymmetrization protocol of linear 1,4-dienes (Figure 14).^35^ A large difference in reactivity between the chiral N,P complex and the diastereotopic sites of the olefin again accounted for the excellent site selectivity in the monohydrogenation. The addition of K_2_CO_3_ is crucial to prevent acid-mediated isomerization of the starting/product allylic alcohols. In the event that the undesired configuration (*syn*)-**22** of the allylic alcohol was formed, a second hydrogenation with the remaining olefin formed the “matched” case and efficiently removed all trace of (*syn*)-**22**. A better understanding of the reactivity differences was provided by kinetic profiling of the hydrogenation using DFT calculations. Once optimized, an array of divinyl carbinols and Boc-protected divinyl carbinamides were efficiently hydrogenated producing the envisioned monohydrogenated alkene in high yields (**22a**–**f**). Included examples consisted of heterocyclic substituents, tertiary divinyl carbinols, functionalized side-chains, and also purely alkyl substituted substrates.

## Applications in Organic Synthesis

The presented methodologies that have either focused on the use of or neutralizing the generated Brønsted acid all yielded a high degree of molecular complexity in a straightforward, clean, and efficient manner starting from relatively simple precursors. To underline the usefulness, the (formal) synthesis of natural products, other biologically relevant compounds and diphosphine ligands are outlined in this section and follow the order as the described methodologies.

### (*S*)-Curcumene

**Figure 15:**
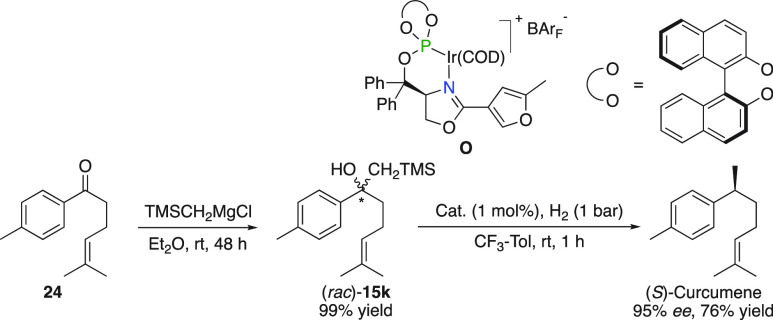
Synthesis of (*S*)-Curcumene.

The advantage of elimination and subsequent hydrogenation of β-hydroxysilanes over unfunctionalized olefins was demonstrated in the preparation of Curcumene, following a simple addition/hydrogenation sequence as depicted in Figure 15.

### Sertraline and PB 28

**Figure 16:**
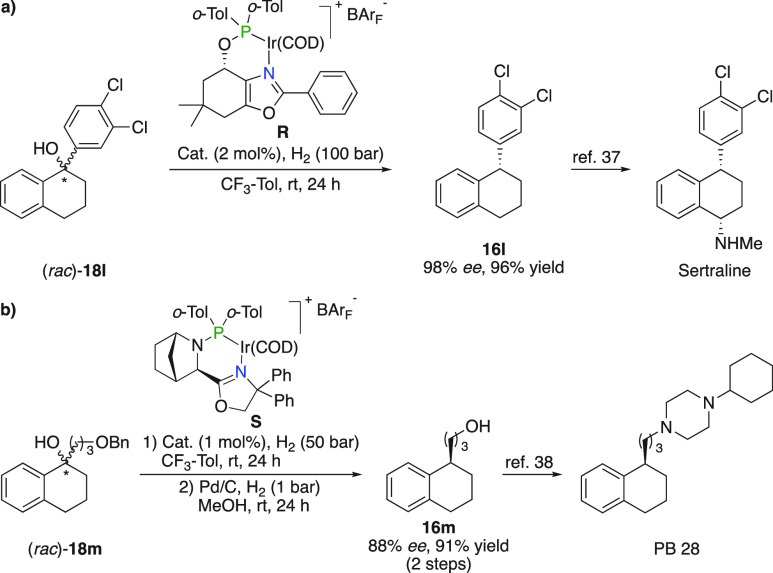
(a) Synthesis of Sertraline. (b) Synthesis of PB 28.

The formal deoxygenation strategy was performed on tertiary alcohol (*rac*)-**18l**, granting access to an intermediate in the synthesis of the antidepressant Sertraline in 98% *ee* (Figure 16a).^37^ In addition, alcohol (*rac*)-**18m** underwent elimination and subsequent hydrogenation yielding **16m** for the synthesis of σ_2_-receptor ligand PB 28 (Figure 16b).^38^

### Diphosphine Ligands

**Figure 17:**
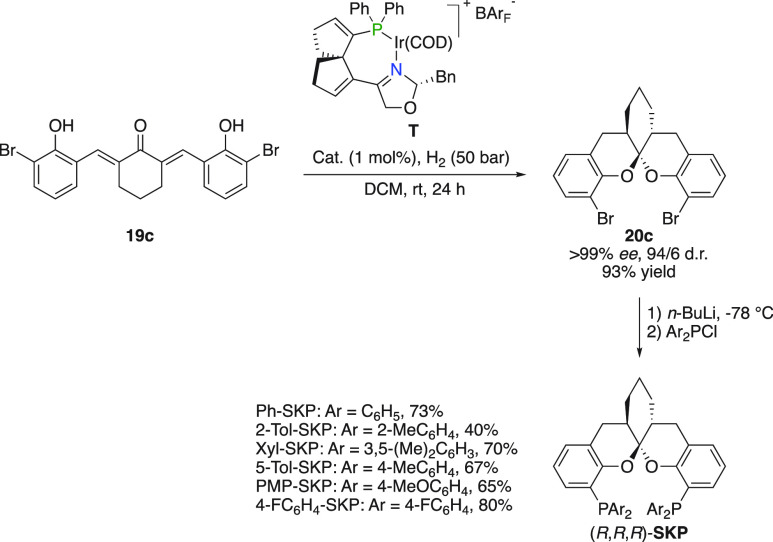
Synthesis of aromatic spiroketal based disphosphine ligands (SKPs).

Dibrominated spiroketal **20c**, prepared by the consecutive hydrogenation and in situ spiroketalization of α,α′-bis(2-hydroxyarylidene) ketone **19c**, was utilized to access a range of privileged aromatic spiroketal based diphosphine ligands (SKPs, Figure 17).^31,39^ An unusually large bite angle, a variable backbone, and large scale accessibility are prominent features of this versatile class of ligands. The SKP ligands have successfully been applied in a number of enantioselective metal-catalyzed reactions.^39b^

### (15*R*)-Pumiliotoxin A

**Figure 18:**
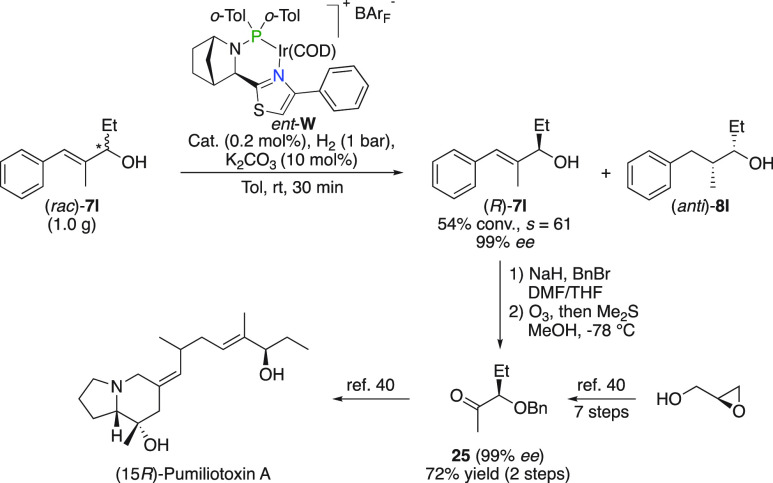
Synthesis of (15*R*)-Pumiliotoxin A.

The developed kinetic resolution was demonstrated to be scalable to gram-scale after which the resolved allylic alcohol (*R*)-**7l** was benzylated to subsequently undergo ozonolysis yielding chiral ketone **25** (Figure 18). This α-chiral ketone constitutes a key intermediate in the total synthesis of the pharmacologically active dendrobatid alkaloid (15*R*)-Pumiliotoxin A, the synthesis of which was previously realized in a 7-step sequence starting from enantiomerically pure glycidol.^40^

### (3*S*)-Inthomycin A and B

**Figure 19:**
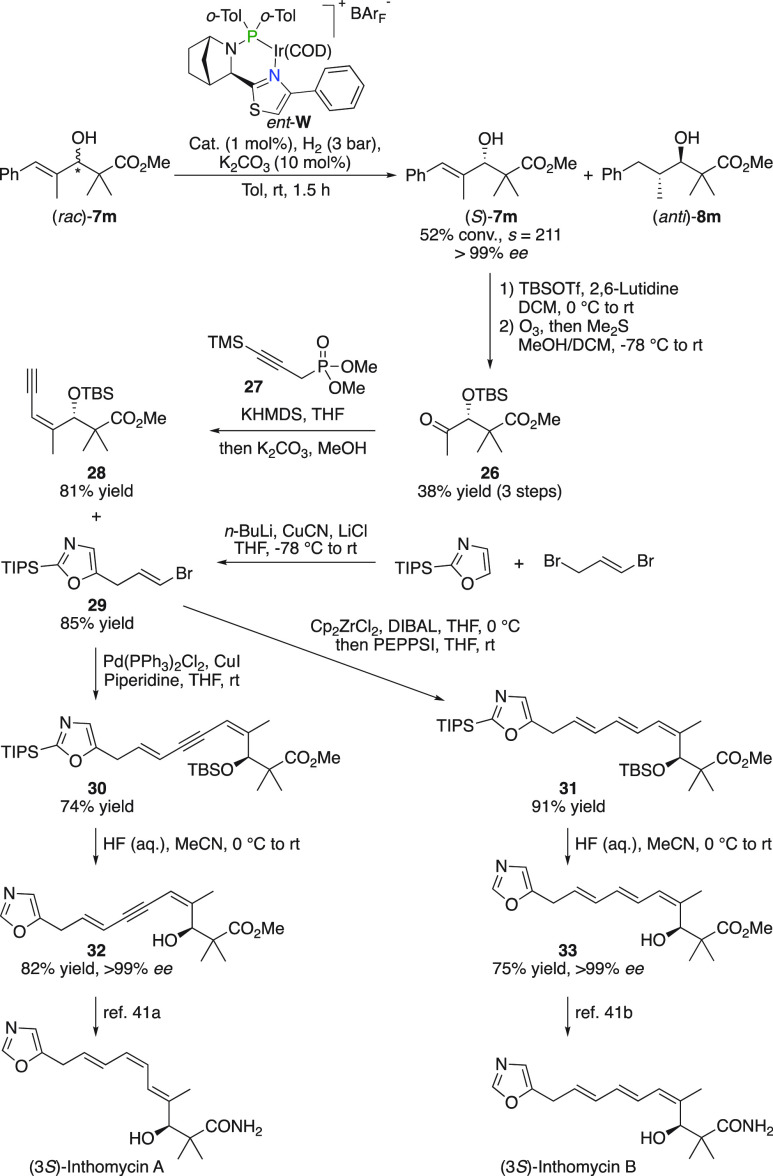
Synthesis of (3*S*)-Inthomycin A and B.

Another application of the hydrogenative kinetic resolution was demonstrated in the synthesis of the Inthomycin family of polyenes (Figure 19). Kinetic resolution of (*rac*)-**7m**, with an outstanding *s*-factor of 211, followed by silylation and ozonolysis afforded chiral ketone **26**. A Horner–Wadsworth–Emmons olefination using phosphinate **27** formed enyne **28** that together with vinyl bromide **29** (prepared from 2-triisopropylsilyloxazole and 1,3-dibromopropene) was coupled in a divergent manner to yield fragments **30** and **31**. Both dienyne **30** and triene **31** were then deprotected using aqueous HF to liberate **32** and **33**, respectively, as a single enantiomer (>99% *ee*). These fragments were converted to either (3*S*)-Inthomycin A or (3*S*)-Inthomycin B according to literature.^41^

### Zaragozic Acid A

**Figure 20:**
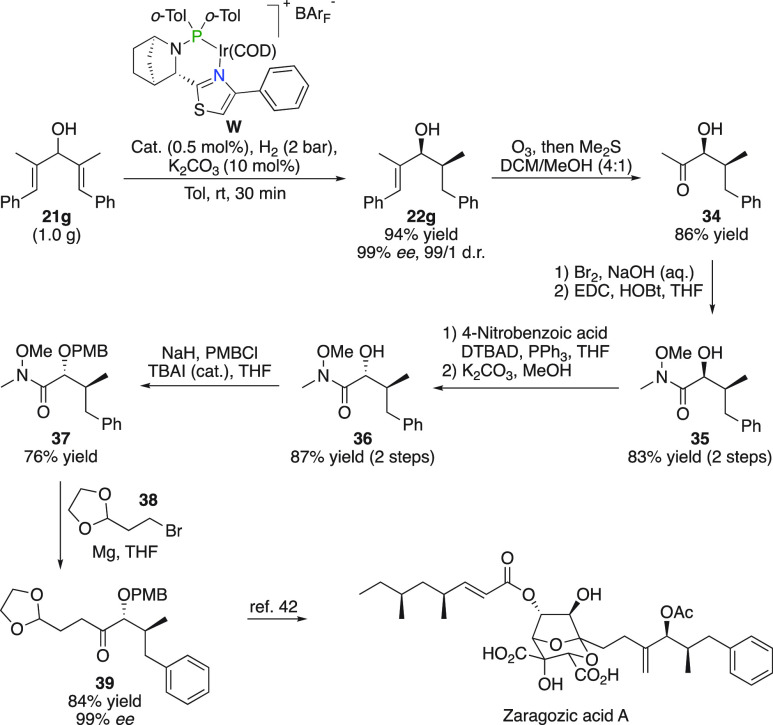
Synthesis of Zaragozic acid A.

Gram-scale desymmetrization was performed on divinyl carbinol **21g** to afford **22g** (Figure 20). Ozonolysis then yielded **34**, after which the methyl ketone was transformed into Weinreb amide **35**. Inversion of the hydroxyl stereochemistry by a Mitsunobu reaction followed by *para*-methoxybenzyl protection allowed a Grignard reaction on **37** to produce **39**, which previously was described as an intermediate in the total synthesis of Zaragozic acid A.^42^

### (+)-Invictolide

**Figure 21:**
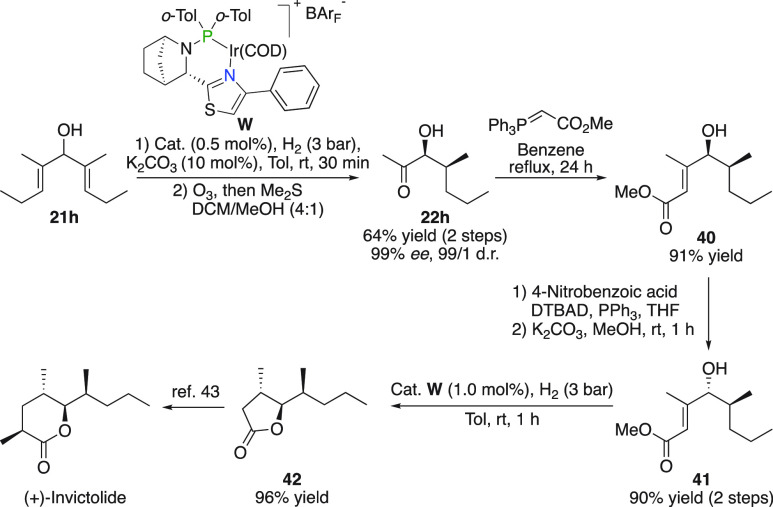
Synthesis of (+)-Invictolide.

The desymmetrization protocol was further illustrated on aliphatic substituted divinyl carbinol **21h** for the synthesis of (+)-Invictolide (Figure 21). After **22h** was obtained by a hydrogenative desymmetrization/ozonolysis sequence, a Wittig–Horner reaction produced α,β-unsaturated methyl ester **40**. Inversion of the carbinol stereochemistry followed by an iridium-catalyzed asymmetric hydrogenation of the remaining olefin yielded lactone **42**, which was cyclized under hydrogenation conditions by the generated Brønsted acid. Butyrolactone **42** represents a reported intermediate in the synthesis of (+)-Invictolide.^43^

## Outlook and Opportunities

The delineated iridium-catalyzed hydrogenations involving a Brønsted acid generated upon decomposition of metal hydride intermediates started with examples in which undesired side-products were formed. Three options are presented in literature to suppress undesired reactions: (1) change of catalyst to employ less acidic complexes, (2) addition of base to buffer the reaction medium (albeit often reported with reduced reactivity), and (3) use of an acid-stable protecting group. Although not explicitly commented on by the authors, protodefluorination due to the complex’s acidity may partly account for the observed defluorinated products in the iridium-catalyzed hydrogenation of alkenyl fluorides.^44^

Perhaps more enticing are catalytic systems that completely neutralize or use the generated Brønsted acid for the development of new methodologies. While a few interesting protocols have emerged in recent years, there is great potential in utilizing the formation of Brønsted acid for further transformations. We anticipate that such new innovations will inevitably continue to emerge in the near future.

As counts for many metal-catalyzed reactions, mechanistic understanding drives design. Little is known about the protonation pathway of substrates, and more mechanistic studies are required to give insight into this process. Protonation most likely occurs via a substrate bound iridium species as already mentioned by Andersson as well as Ding and Wang, although the details and possible extension to other substrates are yet limited.^30,31^

The acid lability of hydroxyl groups is profound in the hydrogenations listed in this Perspective. Andersson demonstrated that oxygen isotopes can be incorporated in the product by the addition of ^18^O-labeled water to the dynamic kinetic resolution of allylic alcohols.^33^ Yet, the use of other returning nucleophiles has not been explored. Matsuda demonstrated the displacement of hydroxyl groups with enoxysilane nucleophiles catalyzed by achiral iridium complexes under a hydrogen atmosphere.^45^ However, the combination of allylic substitution and asymmetric hydrogenation reaction remains unstudied at present.

Building on the idea of intercepting carbocations, an array of other cyclization/hydrogenation sequential reactions (for example Friedel–Crafts or Nazarov cyclization) are still to be explored.

Furthermore, biomass is made up of a large number of potentially labile hydroxyl groups, holding opportunity for valorization processes using H_2_ as a cheap and green reductant.

Lastly, the absolute majority of iridium-catalyzed asymmetric hydrogenations are enantiodivergent in which different olefin geometries of a given olefin are hydrogenated to opposite enantiomers in favor.^28^ The strong Brønsted acidic media during hydrogenation offers the possibility for the development of convergent catalysis via acid-catalyzed isomerization of alkenes. Once accompanied by a large rate difference between both starting materials, hydrogenation of isomeric mixtures can be established in ideality. Aside, olefin migration prior to hydrogenation can potentially lead to the reduction of unreactive olefins. Hydrogenations using deuterium gas are sometimes reported to give substantial amounts of deuterium incorporation in the allylic position, which is suggested to occur as a consequence of olefin migration.^6b,10,46^ Despite that other pathways can also lead to the observed deuterium scrambling, the migration of olefins under hydrogenative conditions remains a topic for further study.^47^

## Dedication

The authors dedicate this Perspective to Prof. David Tanner (DTU, Denmark) on the occasion of his 67th birthday.
